# Construction and quantitative evaluation of China’s smart older adults care policy: a PMC-index model approach

**DOI:** 10.3389/fpubh.2025.1636044

**Published:** 2025-09-24

**Authors:** Yuxin Chen, Yangsen Huang

**Affiliations:** School of Management, Xuzhou Medical University, Xuzhou, China

**Keywords:** smart older adults care system, PMC index model, policy quantification, policy evaluation, China context

## Abstract

Smart older adults care system policies are of great significance for optimizing the allocation of older adults care resources, promoting the innovation and development of smart older adults care system, and effectively responding to population aging. Evaluation of smart older adults care system policy texts can provide theoretical support and decision-making basis for the scientific formulation, effective implementation, adjustment and optimization of smart older adults care system policies. The study analyzes 10 representative policy texts from 72 policies during 2019–2024, and the strengths and weaknesses of each policy and the optimization and adjustment paths are analyzed by calculating the PMC index and drawing PMC surface and radar diagrams. The results show that 3 of the 10 representative policies are assessed as “reasonable and complete,” 6 are rated as “focused,” and 1 is rated as “weak in applicability.” The mean value of PMC index is 7.38. The study shows that the overall design of smart older adults care system policies is scientific, reasonable and of good quality, but there is still room for improvement in many aspects. The performance is as follows: single policy timeliness, short-term policies are more common; diversity of policy aims needs to be enriched; policy content coverage is not comprehensive. Subsequent policies should be improved by focusing on policy timeliness, policy aims and policy content.

## 1 Introduction

At present, the global population has entered an aging phase with accelerating trends. By 2002, the proportion of people aged 65 and above worldwide had exceeded 7%, officially marking the onset of an aging society. By 2022, this demographic group accounted for approximately 9.8% of the total population. In terms of the speed of change, from 2002 to 2012 and 2012 to 2022, the world’s aging rate increased by 0.08 and 0.20% annually respectively, indicating a significant acceleration in the aging process (1). According to the United Nations standard, when a country has 10 percent of its population over 60 years old or 7 percent over 75 years old, it is considered an aging society (2). China’s aging society status was confirmed when its population over the age of 60 accounted for 10% in 2000 (3). Data from China’s National Bureau of Statistics shows that by the end of 2023, the population aged 60 and above constituted 21.1% of the total, with those aged 65 and above accounting for 15.4% (4). Projections suggest that by around 2035, the population aged 60 and above will exceed 400 million, representing over 30% of the total population (5). Population aging has become a crucial trend in China’s social development, characterized by large scale, rapid progression, and significant regional disparities. The intensifying trends of declining birth rates, population aging, empty nest households, and solitary living pose complex challenges for addressing aging issues (6). The massive demographic scale and accelerating pace have generated diverse and urgent demands for older adults care services. Actively tackling population aging has become a vital task for advancing Chinese-style modernization.

Since the World Health Organization (7) put forward the goal of active aging, China has successively issued policies including the National Medium-and Long-Term Plan for Actively Addressing Population Aging, Guidelines on Fully Opening the Older Adults Care Service Market and Enhancing Service Quality, the 13th Five-Year Plan for National Aging Development and Older Adults Care System Construction, and the 14th Five-Year Plan for National Aging Development and Older Adults Care Service System. Guided by top-level design principles, multi-sector collaboration, and dynamic optimization, these policies have actively responded to and formed concrete frameworks (8). In recent years, advanced technologies such as internet, big data, cloud computing, and smart devices have been widely applied throughout older adults care services, significantly driving industry development and forming the smart older adults care system sector. Smart older adults care system uses the information management platform to break through the limitations of space and time, and closely connects the daily life of the older adults, such as transportation, health care, daily consumption, entertainment and leisure, etc., which not only saves time and cost and improves the efficiency of services, but also provides personalized services to meet the diversified needs of the older adults, and matches the supply and demand of older adults care resources (9). With the aging population on the rise, smart older adults care system has increasingly received the attention of the government, the market and the society, and has become the development direction for them to solve the problems of the older adults care together. Especially for the government and society, smart older adults care system has become an important orientation of the corresponding policy formulation, and from the central to the local government specific implementation policies have also been formulated (10).

However, smart older adults care system has not yet a completely unified authoritative industry standard and service specification. Although there are many policies on smart older adults care system, there is a certain degree homogeneity in content, mostly focusing on service content, lacking clear instructions and operational standards for access qualification, quality supervision, risk prevention, dispute handling, etc., and the main reasons are the insufficient role of policy guidance, the imperfect policy system, the need to standardize the policy implementation process, and the lack of richness and balance of policies (11). Moreover, the two-sided nature of network technology and digital technology coupled with the complexity of society has brought many variable factors to the development of smart older adults care system, leading to a large number of uncertainty risks. In particular, the use of big data, cloud computing and other cutting-edge technologies will not only trigger the “digital divide,” “information cocoon” and other issues, but also the hidden false information, pseudo-scientific information, etc. may seriously jeopardize the personal safety of the older adults, such as being easily seduced by false investment, leading to economic losses; false medical information leads to old people choose harmful drugs and the wrong treatment method. The dual risks of smart older adults care system policies and their environment make it impossible to maximize the effectiveness of the current policy, and it may directly or indirectly affect the effectiveness of social governance in aging China (10). Therefore, it is necessary to comprehensively assess and analyze China’s smart older adults care system policies and their implementation effects in the current social context, to help identify the problems of existing policies, and to facilitate the adjustment and improvement of subsequent policies, and then improve the governance effect of the policy.

Policy evaluation is crucial for governments to grasp the implementation effects of public policies. Scientifically assessing whether policies possess rationality, integrity, and feasibility provides essential theoretical and practical foundations for subsequent policy formulation and optimization. The evaluation process is influenced by multiple factors, leading to dynamic changes in content and outcomes. Therefore, to ensure the accuracy and interpretability of evaluation results, it is imperative to select objective and scientific evaluation methods (12). While addressing the core issue of “older adults care service shortages,” the smart older adults care system policies must balance multiple objectives including technological accessibility, cost control, and social participation, which may create inherent tensions. The policy requires dynamic coordination across dimensions such as technology application, service provision, and institutional safeguards, with continuous iterative optimization based on evolving social demands. However, the complex influencing factors arising from regional disparities and demographic differences significantly increase the complexity of scientific policy evaluation. Among existing policy evaluation methods, the PMC index model is widely recognized both domestically and internationally as a scientifically rigorous and effective approach for evaluating policy texts (13). For the multidimensional, dynamic, and goal-diverse characteristics of the smart older adults care system policy, the PMC model demonstrates significant methodological advantages. This model can analyze internal consistency in any policy, conduct scientific quantitative evaluations, and identify policy strengths and weaknesses. The study evaluates the rationality and feasibility of policies by conducting research on policy proposals and compiling findings into research reports, thereby guiding policymakers to adjust existing policies or issue new ones (14). Its core advantage lies in establishing a flexible multi-dimensional indicator system that dynamically adjusts evaluation criteria according to policy changes, avoiding the limitations of single-perspective analysis while capturing real-time policy adjustment logic and adaptability effects. However, singular quantitative analysis struggles to deeply explore policy essence. Therefore, this paper integrates SWOT analysis, text analysis tools, and PMC index models for qualitative and quantitative research on smart older adults care system policies. Qualitative analysis identifies strategic logic, dynamic relationships, and value orientations within smart older adults care system policies, while quantitative evaluation via PMC ensures measurable, comparable, and optimizable policy quality. Ultimately, this establishes a comprehensive analytical framework— “macro-level direction, micro-level data, and actionable improvement paths,” providing more thorough and reliable decision support for scientific formulation, precise optimization, and effective implementation of smart older adults care system policies (15).

Based on this, this paper first analyzes the internal and external environments of smart older adults care system using the SWOT framework to identify factors affecting its industrial development. Subsequently, through text data mining of China’s smart older adults care system policies, a quantitative evaluation framework based on the PMC index model was constructed to systematically assess the implementation effectiveness of these policies. To further understand the details and impacts of smart older adults care system policies, as well as their differences and existing issues, 10 representative smart older adults care system policies were selected for empirical analysis. This comprehensive evaluation of their strengths and weaknesses during implementation provides new references and recommendations for adjusting China’s smart older adults care system policies.

The rest of the paper is structured as follows: Section 2 reviews the existing research and literature. Section 3 constructs the PMC index model and designs the variables. Section 4 analyzes the 10 selected policies quantitatively and qualitatively for PMC index calculation. Section 5 discusses the results. Section 6 summarizes the full paper.

## 2 Literature review

With the accelerating global aging population and rapid technological innovation, smart older adults care system integrates information technology to provide multi-scenario older adults care models. As a new direction in global older adults care development, smart older adults care system has attracted extensive academic attention and discussion (16).

Recent years have seen comprehensive research on smart older adults care system covering various aspects. Scholars worldwide have explored multiple dimensions including the evolution of smart older adults care system, innovative service models, and key technologies supporting older adults care systems (17). Scholars both domestically and internationally have extensively studied the development of smart older adults care system. By analyzing the evolution of smart older adults care system applications and evaluating their policy environments, they have assessed the developmental stages, acceptance levels, and accessibility of smart older adults care system programs, elucidating their opportunities and challenges (9, 47). Regarding older adults care models, domestic research has progressed from home-based care and integrated medical-care systems to community-based institutional care, rural care (11) and financial mechanism-based care (18), integrating these approaches to explore optimal solutions. Zhang and Xu (19) developed an intelligent older adults care model driven by primary healthcare, addressing outdated information platforms and the “digital divide” to provide a viable approach for improving home-based care quality. International scholars like Sandberg, Olsson, Gjevjon and Borglin (20) explored older adults-related care models in long-term care environments, paving the way for future interventions that meet seniors ‘essential needs. Lommi et al. (21) proposed the TEC-MED model, which addresses complex care demands through cross-service inclusivity, personalization, and integration. Regarding smart older adults care system technology applications, comprehensive domestic and international research reveals scholars’ focus on smart aging solutions and digital divide challenges. Researchers aim to bridge this gap through technological innovation, promote age-friendly transformations, and help seniors benefit from technological dividends. Domestic studies emphasize IoT and AI applications to enhance service accessibility, building integrated systems that synergize community-based, home, and smart care services (22–24). The ultimate goal is to improve service efficiency and quality to meet diverse older adults needs. Meanwhile, foreign research focuses on precision health data analysis and predictive modeling to optimize daily activities, aiming to boost quality of life and independence. For instance, Shaikh, Dar and Sofi (25) utilized Extended Reality (XR) technology to provide remote medical consultations for older adults individuals. Franco, Condon, Martínez and Ahmed (26) proposed the SEDAR system, which monitors electrical appliances of seniors living alone through smart energy data and machine learning, enabling early detection of home health abnormalities and facilitating remote older adults care services.

Furthermore, with the introduction of a series of policies related to smart older adults care system, Chinese scholars have increasingly focused on studying these policies. Their research explores the development history, existing challenges, shortcomings, and implementation progress of policies, aiming to better promote the evolution and implementation of older adults care service policies (8). Scholars have analyzed policy dimensions from multiple perspectives, including policy actors, policy objectives-policy instruments-policy stages, revealing structural imbalances in the actual use of policy instruments and a preference for environmental and supply-oriented policy tools (27). Additionally, the focus of policy objectives varies across different implementation phases. However, there is little comprehensive and systematic review of smart older adults care system policy from central to local level in academia.

In the development of smart older adults care system policies, researchers have employed various methodologies and models for policy evaluation. For instance, Yue (28) conducted a textual analysis of provincial pension policies from a policy instrument perspective, exploring their implementation status. Zhao, Jiang, Zhang and Liang (29) utilized the Smith model to examine practical challenges in community-based smart older adults care system policies through four dimensions: policy core, implementing agencies, target groups, and environmental factors, proposing optimization strategies. Li and Zhang (30) analyzed China’s smart older adults care system policy implementation challenges using the Mit-Horn policy implementation model, identifying execution bottlenecks and suggesting advancement strategies. Chen and Li (31) conducted quantitative analysis on 21 selected smart older adults care system policies in Hainan Province from 2017 to 2023 through content analysis, revealing existing issues and proposing optimization measures. Liu and Wang (32) processed central-level smart older adults care system policy documents through text cleaning and coding, employing bibliometric and content analysis methods to propose improvement pathways. Few studies have used the PMC index model for quantitative analysis. While these approaches effectively evaluate policies, they all have inherent limitations. For example, policy bibliometrics relies on comprehensive data, whereas textual analysis faces challenges in contextual understanding and data quality. The PMC index model has gained widespread application across disciplines like economics, environmental science, and sociology due to its significant advantages. For instance, Liu, Jia and Xia (12) used the PMC model for dynamic evaluation of new energy vehicle policies. Kuang et al. (14) applied the PMC index model to quantitatively assess China’s land conservation policies, ensuring their sustainable and efficient implementation. Moreover, the PMC index model can avoid the problems existing in previous policy quantitative research, such as limited indicator quantity, strong subjectivity of evaluation, and insufficient accuracy of results. It has the advantages of low cost, strong objectivity, and high precision (33). Therefore, this paper selects the PMC index model to conduct quantitative evaluation on China’s smart older adults care system policy.

The selection of policy evaluation methods directly impacts the accuracy of assessment outcomes. Relying solely on qualitative or quantitative approaches may lead to biased results. Integrating multiple research methods such as qualitative and quantitative analysis with complementary strengths enables comprehensive and objective policy evaluations (34). Building on this foundation, this study combines qualitative and quantitative approaches: applying the SWOT method for internal and external environment analysis of smart older adults care system policies, conducting text mining using ROSTCM6, and implementing the PMC index model for policy evaluation. This approach aims to bridge the current gap in quantitative research within the field, objectively assess the strengths and weaknesses of existing policies, and propose practical optimization strategies to address current challenges.

## 3 Research design

### 3.1 Data sources and processing

Since 2019, the research and construction of smart older adults care system have been gradually emphasized and promoted, and more and more related policies have been issued by the government. In order to make the collected policy data more accurate, reliable, complete and representative, this paper selected the policies released in the period of 2019–2024, covering the central, provincial and municipal levels, and searched the database of Peking University Laws and Regulations Database and White Deer Think Tank with the keywords of “Smart Older Adults Care Service” and “Smart+Older Adults Care Service,” and the policy texts with smart older adults care system as the core theme were selected, while those with little relevance to the theme of older adults service, those that were invalid, and those that had not yet been implemented were deleted. In order to make the analysis more rigorous, this paper also used policies promulgated on the official websites of all levels of governments as benchmarking materials to compare, validate and supplement their sources and contents. In the end, 72 policy texts with strong relevance and validity to smart older adults care system were obtained. Of these, 4 are at the central level, 17 at the provincial level, and 51 at the municipal level.

### 3.2 Construction of PMC indicator model

PMC index model (Policy Modeling Consistency Index) was established by Estrada (35), and its main idea comes from his “Omnia Mobilis” hypothesis, that is, every variable can not be ignored. Through mining the text, combining the characteristics of policies to construct the evaluation index system, exploring the interconnection between the variables, the model constructs the primary and secondary variables, which are not limited in number and each secondary variable has the same weight. The binary algorithm is used to calculate the PMC index, which intuitively reflects the advantages and disadvantages and consistency of policies (36). By quantifying policies, the PMC index model transforms abstract concepts in policy texts into computable numbers, making research more scientific and objective, and reducing artificial emotional interference and decision-making bias (13), so as to better formulate and implement policies. Therefore, this paper uses the PMC index model to evaluate and optimize the adjustment of smart older adults care system policies. The PMC model in this paper is divided into five steps (37): (1) Policy text collection and preprocessing; (2) Index system construction; (3) PMC index calculation; (4) PMC surface plotting; (5) Comprehensive policy analysis.

### 3.3 Word frequency analysis and variable design

#### 3.3.1 Word frequency analysis

**Figure 1:**
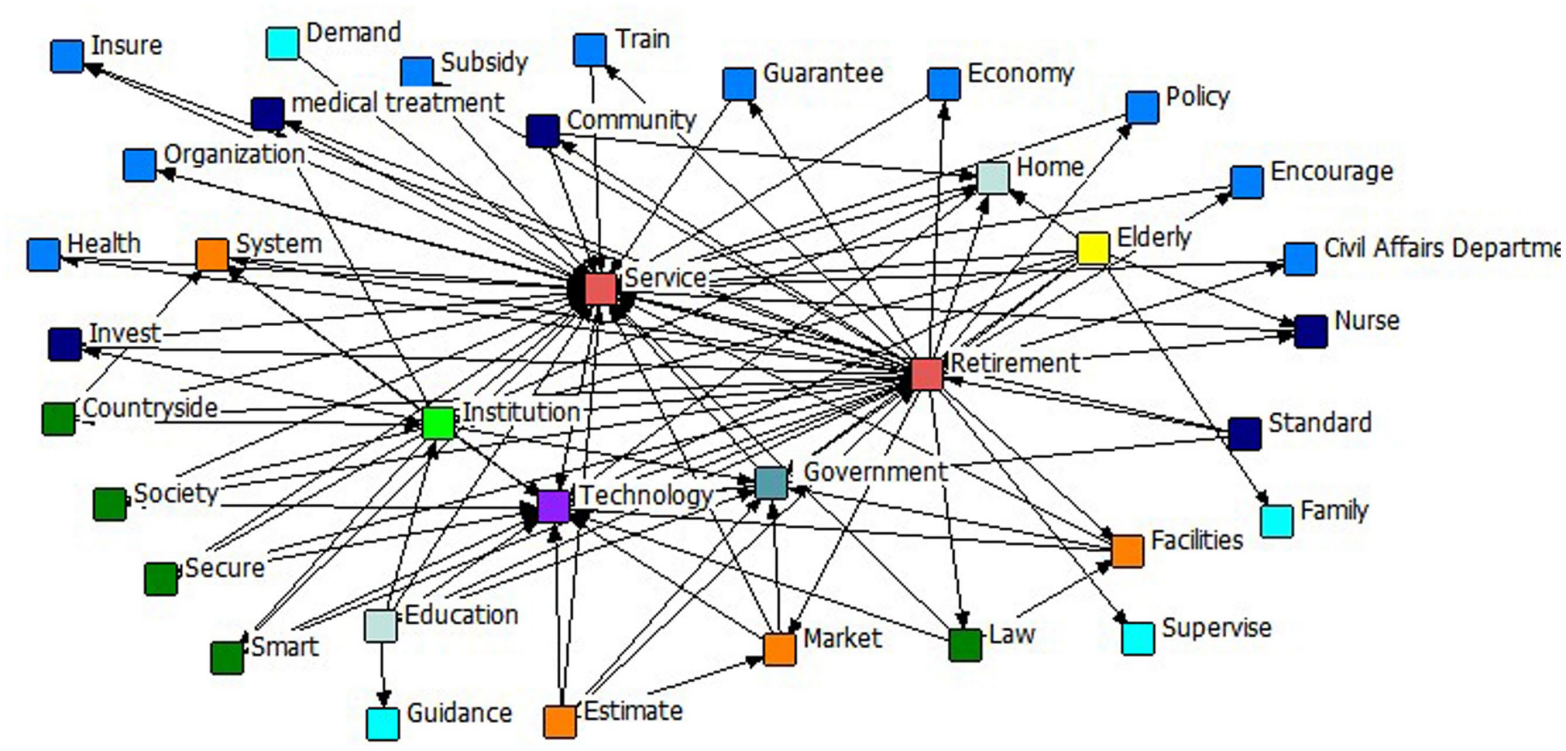
Semantic network graph.

Analyzing and mining policy texts helps to gain a deeper understanding of the key content of policies, thus targeting, scientifically and systematically identifying the core focus of policies and helping policymakers to more accurately select assessment indicators and design relevant variables. Through deep mining of policy texts, it can provide more specific reference bases for policy evaluation and subsequent decision-making, and help ensure that the implementation and effects of policies meet the expected goals. In this study, 72 policy texts were first integrated into a TXT file and then imported into ROSTCM6 for semantic network analysis. Since the text contains words without practical meaning such as “above,” “should,” “first,” etc., manual processing is needed to filter out and delete these words. Then through the semantic network analysis, the association between the policy feature words can be presented, and the core nodes between the main feature words in the text can be identified intuitively. Figure 1 below shows the semantic network graph generated by semantic network analysis of 72 items of text data using ROSTCM6 analysis software. The semantic network graph presents the key nodes and associations in the 72-item text, from which the degree of synergy between the policy contents can be analyzed. In the network graph, each node represents a keyword, and the connecting lines between nodes indicate the co-occurrence relationship between these keywords. The size of the nodes and the connecting lines between the nodes reflect the hotness of the keywords and the degree of connection between them. The closer a keyword is to the center of the network graph, the more connecting lines it radiates, which indicates the higher importance of that keyword (38). From the semantic network graph, it can be seen that “Retirement” and “Service” are located in the core position, with more lines and a greater degree of connection between them and other keywords, indicating that smart older adults care system policy topics are relatively concentrated.

#### 3.3.2 Variable design

This study integrates Estrada’s variable design methodology with existing research findings to identify first-level indicators demonstrating strong cross-research universality, establishing an initial framework that encompasses dimensions such as policy nature, jurisdictional scope, implementation timeline, target demographics, and operational tools. Building on the characteristics of smart older adults care system policies, we supplemented this framework through semantic network mapping and analysis of high-frequency policy terms. This expansion introduces secondary variables including policy content, implementation measures, evaluation metrics, and strategic objectives (19, 39–41), while maintaining the foundational structure for policy assessment. The enhanced framework significantly improves explanatory power in interpreting smart older adults care initiatives, effectively bridging theoretical foundations with practical applications.

According to the principles of purposefulness, completeness, operability, independence, significance and dynamism in the design process of the index system, in this study, we set 9 primary variables and 33 secondary variables. The primary variables are Policy Nature $X_{1}$, Policy Area $X_{2}$, Policy Timeliness $X_{3}$, Policy Aim $X_{4}$, Policy Content $X_{5}$, Policy Object $X_{6}$, Policy Evaluation $X_{7}$, Policy Measure $X_{8}$ and Policy Tool $X_{9}$ as shown in Table 1. Drawing from previous research practices, to ensure consistent weighting across secondary variables in the evaluation system, a binary scoring method is adopted. Specifically, when policy text content aligns with variable criteria, it is assigned a value of 1; otherwise, it is marked as 0. The operational procedure involves: Researchers review policy texts item by item based on predefined clear standards. If the text explicitly meets or encompasses specific definition requirements for a variable, that variable receives a score of 1. Conversely, if the text fails to mention or inadequately addresses the variable’s definition criteria, it is uniformly assigned a score of 0.

**Table 1:** Variable design.

Primary variables	Secondary variables	Evaluation criteria for secondary variables
Policy Nature $X_{1}$	Supervision $X_{11}$ Prediction $X_{12}$ Guidance $X_{13}$	Judge whether the policy involves supervision in development. If it is, then it scores 1; if not, then it scores 0. Judge whether the policy involves a description of future development results. If it is, then it scores 1; if not, then it scores 0. Judge whether the policy involves the guiding goals of development. If it is, then it scores 1; if not, then it scores 0.
Policy Area $X_{2}$	Economy $X_{21}$ Medicine $X_{22}$ Technology $X_{23}$	Judge whether the policy involves an economic area. If it is, then it scores 1; if not, then it scores 0. Judge whether the policy involves a medical area. If it is, then it scores 1; if not, then it scores 0. Judge whether the policy involves a technology area. If it is, then it scores 1; if not, then it scores 0.
Policy Timeliness$X_{3}$	long term$X_{31}$ mid-term$X_{32}$ short-term $X_{33}$	Judge whether the policy covers more than 5 years. If it is, then it scores 1; if not, then it scores 0. Judge whether the policy covers 3–5 year content. If it is, then it scores 1; if not, then it scores 0. Judge whether the policy covers up to 3 years. If it is, then it scores 1; if not, then it scores 0.
Policy Aim $X_{4}$	Product development $X_{41}$ Publicity and Promotion $X_{42}$ Service Supply Optimization $X_{43}$	Judge whether the policy aim involves the development of products that meet the needs of the older adults. If it is, then it scores 1; if not, then it scores 0. Judge whether the policy aim involves publicizing and promoting older adults services. If it is, then it scores 1; if not, then it scores 0. Judge whether the policy aim involves optimizing service provision. If it is, then it scores 1; if not, then it scores 0.
Policy Content$X_{5}$	Application Pilot $X_{51}$ Technical Training $X_{52}$ Government Subsidy $X_{53}$ Smart Device $X_{54}$ Network Security $X_{55}$	Judge whether the policy involves the content of application pilot. If it is, then it scores 1; if not, then it scores 0. Judge whether the policy involves the content of technical training or education for the older adults. If it is, then it scores 1; if not, then it scores 0. Judge whether the policy involves the content of government subsidies. If it is, then it scores 1; if not, then it scores 0. Judge whether the policy involves the content of smart devices. If it is, then it scores 1; if not, then it scores 0. Judge whether the policy involves the content of network security. If it is, then it scores 1; if not, then it scores 0.
Policy Object $X_{6}$	Government $X_{61}$ Business Unit $X_{62}$ Community $X_{63}$ Household $X_{64}$	Judge whether the policy object involves government. If it is, then it scores 1; if not, then it scores 0. Judge whether the policy object involves business units. If it is, then it scores 1; if not, then it scores 0. Judge whether the policy object involves community. If it is, then it scores 1; if not, then it scores 0. Judge whether the policy object involves household. If it is, then it scores 1; if not, then it scores 0.
Policy Evaluation $X_{7}$	Sufficient Basis $X_{71}$ Planning Details $X_{72}$ Scheme Science $X_{73}$	Judge whether the policy basis is sufficient. If it is, then it scores 1; if not, then it scores 0. Judge whether the policy is planned in detail. If it is, then it scores 1; if not, then it scores 0. Judge whether the scheme of the policy is scientific. If it is, then it scores 1; if not, then it scores 0.
Policy Measure $X_{8}$	Institutional Reform $X_{81}$ Financial Support $X_{82}$ Cultivation of Talent $X_{83}$ Legal Safeguards $X_{84}$ Assessment and Evaluation $X_{85}$	Judge whether the policy involves institutional reforms to build specialized institutions for the older adults. If it is, then it scores 1; if not, then it scores 0. Judge whether the policy involves financial support. If it is, then it scores 1; if not, then it scores 0. Judge whether the policy involves talent training. If it is, then it scores 1; if not, then it scores 0. Judge whether the policy involves legal safeguards. If it is, then it scores 1; if not, then it scores 0. Judge whether the policy involves assessment l and evaluation. If it is, then it scores 1; if not, then it scores 0.
Policy Tool $X_{9}$	Compulsory Type $X_{91}$ Innovative Type $X_{92}$ Market Type$X_{93}$ Cooperative Type $X_{94}$	Judge whether the policy specifies that certain behaviors are prohibited or permitted. If it is, then it scores 1; if not, then it scores 0. Judge whether the policy involves the integration of technological and institutional innovation or industrial innovation. If it is, then it scores 1; if not, then it scores 0. Judge whether the policy gives full play to the role of market resource allocation. If it is, then it scores 1; if not, then it scores 0. Judge whether the policy involves international cooperation. If it is, then it scores 1; if not, then it scores 0.

### 3.4 Construction of multi-input–output table

Multi-input–output table is a kind of data analysis framework formed by multidimensional measurement of variables, which has the same weight for several secondary variables set under each primary variable (42). According to the evaluation system, each secondary variable is assigned a binary value, which is 1 if the policy text contains the content of the relevant secondary variable and 0 otherwise, and the value range of the primary variable is [0,1]. Table 2 below shows the multi-input–output table we constructed.

**Table 2:** Multi-input–output table.

Primary variables	Secondary variables
$X_{1}$	$X_{11}X_{12}X_{13}$
$X_{2}$	$X_{21}X_{22}X_{23}$
$X_{3}$	$X_{31}X_{32}X_{33}$
$X_{4}$	$X_{41}X_{42}X_{43}$
$X_{5}$	$X_{51}X_{52}X_{53}X_{54}X_{55}$
$X_{6}$	$X_{61}X_{62}X_{63}X_{64}$
$X_{7}$	$X_{71}X_{72}X_{73}$
$X_{8}$	$X_{81}X_{82}X_{83}X_{84}X_{85}$
$X_{9}$	$X_{91}X_{92}X_{93}X_{94}$

### 3.5 Calculation of PMC index and evaluation grade classification

The calculation of PMC index can be divided into the following three steps according to the following formula, firstly, assigning values to the 33 secondary variables according to Equations (1, 2); then calculating the scores of 9 primary variables, respectively, according to Equation (3); and finally, adding up the primary variables of each policy to arrive at the PMC index of the policy according to Equation (4).


(1) X~N[0,1]

(2) $$X=\{X_{R}:[0,1]\}$$

(3) $$X_{i}=[\sum_{j=1}^{n}\frac{X_{i-j}}{T(X_{i-j})}],i=1,2,\ldots ,n\;$$

(4) $$\mathrm{PMC}=[\begin{array}{c} X_{1}\sum_{j=1}^{3}\frac{X_{1-j}}{3}+X_{2}\sum_{j=1}^{3}\frac{X_{2-j}}{3}+X_{3}\sum_{j=1}^{3}\frac{X_{3-j}}{3}\\ X_{4}\sum_{j=1}^{3}\frac{X_{4-j}}{3}+X_{5}\sum_{j=1}^{5}\frac{X_{5-j}}{5}+X_{6}\sum_{j=1}^{4}\frac{X_{6-j}}{4}\\ X_{7}\sum_{j=1}^{3}\frac{X_{7-j}}{3}+X_{8}\sum_{j=1}^{5}\frac{X_{8-j}}{5}+X_{9}\sum_{j=1}^{4}\frac{X_{9-j}}{4} \end{array}]\;$$

Where *i* denotes a primary variable, *j* denotes a secondary variable, and *n* is the number of secondary variables corresponding to the primary variable *i*, *j*, *n* = 1, 2, 3, 4,….

PMC index model is a kind of narrow policy evaluation, for the classification of PMC evaluation level, if we directly judge the advantages and disadvantages of the policy, such as “perfect,” “excellent,” “qualified,” these words are rough and easy to cause misunderstanding in a broad sense (37). Therefore, the classification criteria of PMC index evaluation grade are shown in Table 3 below.

**Table 3:** Evaluation.

PMC index score	Grade division
PMC≤ 6.50	Weak applicability
6.50< PMC≤ 7.50	Focused
PMC> 7.50	Reasonable and Complete

### 3.6 Construction of PMC surface diagrams

The PMC surface plot visualizes the PMC index table, which can show the degree of strengths and weaknesses of the policy on each variable in a more intuitive and all-round way than the PMC index, and helps to analyze the strengths and weaknesses of the smart older adults care system policies (36). The hierarchical level of policy samples can be assessed through the concavity of their surface diagrams, which reflects internal coordination consistency within policies. A smaller concavity indicates higher scores and better internal coordination at that indicator level, while a larger concavity suggests lower scores and potential issues in policy implementation. In this paper, the 9 primary variables are built according to equation (5) to establish a 3×3 PMC surface matrix and plot the surface diagram.


(5) $$\mathrm{PMC}-\text{surface}=[\begin{array}{ccc} X_{1} & X_{2} & X_{3}\\ X_{4} & X_{5} & X_{6}\\ X_{7} & X_{8} & X_{9} \end{array}]$$

## 4 Data processing and results of smart older adults care system policies

### 4.1 Choice of representation policies

In order to make the selection of representative policy samples comprehensive and integrated, this paper combines central and local level considerations, and, after text mining, selects 10 representative smart older adults care system policies in a ratio of 2:4:4 among the 72 policy documents selected at the central, provincial, and municipal levels. Regarding the selection at the central level, we choose the more comprehensive and instructive first-level programmatic policy documents. For the selection at the local level, considering the differences between provinces and provinces, municipalities and municipalities, and provinces and municipalities, we selected the guiding documents issued by the people’s congresses, their standing committees and people’s governments of various provinces and cities, which were from the eastern and central regions with different levels of economic development and could systematically reflect the local practice characteristics. Among the selection of policies at the provincial level, this paper selects policy texts from Anhui Province, Zhejiang Province, Hainan Province and Shandong Province. In the selection of municipal policies, this paper selects Yangzhou City, two municipalities-Tianjin and Shanghai, and Zhuhai Special Economic Zone, which facilitates the comparison of the differences in smart older adults care system policies in regions with different economic conditions. Based on the analysis from both national and local perspectives, this paper aims to systematically identify the goal-setting characteristics of central and local policies, simultaneously reveal the differentiated pattern of regional practices, and refine the governance path with universality and adaptability for policy optimization. Based on the PMC index model, these 10 smart older adults care system policies are comprehensively evaluated to deeply explore the specific content of the policies, and then provide reference and new ideas for future policy making. As shown in Table 4 below.

**Table 4:** Smart older adults care system representative policies.

Policy code	Policy name	Formulate organ	Release time
P1	National Plan for the Development of the Aging Career and the Older Adults Service System in the 14th Five-Year Plan	State Council (PRC)	2021.12.30
P2	Opinions on Promoting the Development of Older Adults Services	State Council Office of the People’s Republic of China	2019.04.16
P3	Regulations on Older Adults Services in Anhui Province	Anhui Provincial People’s Congress (including Standing Committee)	2022.09.29
P4	Regulations on the Promotion of Social Services for the Older Adults in Zhejiang Province	Zhejiang Provincial People’s Congress (including the Standing Committee)	2021.09.30
P5	Regulations on Older Adults Services in Hainan Province	Hainan Provincial People’s Congress (including Standing Committee)	2023.05.24
P6	Shandong Province’s “14th Five-Year Plan” for Old-Age Service Systems	Shandong Provincial People’s Government	2021.07.29
P7	Regulations on Home-based Older Adults Services in Yangzhou City	Yangzhou Municipal People’s Congress (including the Standing Committee)	2021.06.08
P8	Regulation on the Promotion of Older Adults Services in Tianjin	Tianjin Municipal People’s Congress (including Standing Committee)	2020.12.01
P9	Regulations on Older Adults Services in Shanghai	Shanghai Municipal People’s Congress (including Standing Committee)	2023.12.18
P10	Regulations on the Promotion of Home-based Older Adults Services in the Zhuhai Special Economic Zone	Zhuhai People’s Congress (including Standing Committee)	2022.12.02

In order to consider the comprehensiveness of the smart older adults care system issue in the research and analysis, this paper firstly uses SWOT to systematically analyze the strengths and weaknesses, opportunities and threats of the internal and external environment of the smart older adults care system service industry. The strengths of smart older adults care system lie in the fact that it reduces the stress of family members, provides precise services, enriches the lives of the older adults, improves the efficiency of older adults care and promotes the construction of a healthy China. However, smart older adults care system also has shortcomings such as expensive construction costs, lack of professionals in the field of product development, digital divide, and privacy leakage. In today’s social trend, high market demand, national policy support, technological progress, development of convergence industry, and international cooperation bring opportunities for smart older adults care system, while at the same time, there are also threats such as competition among organizations, too small scope of service of the platform, decentralized demonstration pilots, difficulty in forming industrial scale, and insufficient promotion efforts.

### 4.2 PMC indices for 10 representative policies

This paper determined the scores of each secondary variable by text mining and content analysis of the 10 policy texts and substituting them into the multiple input–output table, as shown in Table 5. Second, Equation (4) was applied to further calculate the score of the PMC index of each smart older adults care system policy (the results were retained in two decimal places) and rank and classify the size of the score, as shown in Tables 6, 7.

**Table 5:** Input–output table for 10 policies.

Primary variables	Secondary variables	P1	P2	P3	P4	P5	P6	P7	P8	P9	P10
$X_{1}$	$X_{11}$ $X_{12}$ $X_{13}$	1 1 1	1 1 1	1 0 1	1 0 1	1 0 1	1 1 1	1 0 1	1 0 1	1 0 1	1 0 1
$X_{2}$	$X_{21}$ $X_{22}$ $X_{23}$	1 1 1	1 1 1	1 1 1	1 1 1	1 1 1	1 1 1	1 1 1	1 1 1	1 1 1	1 1 1
$X_{3}$	$X_{31}$ $X_{32}$ $X_{33}$	1 1 0	0 1 1	0 0 1	1 0 0	0 0 1	0 1 1	1 0 1	0 0 1	1 0 1	0 0 1
$X_{4}$	$X_{41}$ $X_{42}$ $X_{43}$	1 1 1	1 0 1	1 1 1	0 0 1	1 0 1	1 1 1	0 1 1	0 1 1	1 1 1	0 1 1
$X_{5}$	$X_{51}$ $X_{52}$ $X_{53}$ $X_{54}$ $X_{55}$	1 1 1 1 1	0 1 1 1 0	1 1 1 1 0	0 1 1 1 0	0 1 1 1 0	1 1 1 1 0	0 1 1 1 0	0 1 1 1 0	0 1 1 1 0	1 1 1 1 0
$X_{6}$	$X_{61}$ $X_{62}$ $X_{63}$ $X_{64}$	1 1 1 1	1 1 1 1	1 1 1 1	1 1 1 1	1 1 1 1	1 1 1 1	1 1 1 1	1 1 1 1	1 1 1 1	1 1 1 1
$X_{7}$	$X_{71}$ $X_{72}$ $X_{73}$	1 1 1	1 1 1	1 1 1	1 1 1	1 1 1	1 1 1	1 1 1	1 1 1	1 1 1	1 1 1
$X_{8}$	$X_{81}$ $X_{82}$ $X_{83}$ $X_{84}$ $X_{85}$	1 1 1 1 1	1 1 1 0 1	0 1 1 1 1	0 1 1 1 1	0 1 1 1 1	1 1 1 1 1	0 1 1 1 1	0 0 1 1 1	1 1 1 1 1	0 1 1 1 1
$X_{9}$	$X_{91}$ $X_{92}$ $X_{93}$ $X_{94}$	1 1 1 1	1 1 1 0	1 1 1 0	1 0 1 0	1 1 1 1	1 1 1 1	1 0 1 0	1 1 1 0	1 1 1 1	1 1 1 0

**Table 6:** PMC index scores for 10 representative policies.

Primary variables	P1	P2	P3	P4	P5	P6	P7	P8	P9	P10	Average value
$X_{1}$	1.00	1.00	0.67	0.67	0.67	1.00	0.67	0.67	0.67	0.67	0.77
$X_{2}$	1.00	1.00	1.00	1.00	1.00	1.00	1.00	1.00	1.00	1.00	1.00
$X_{3}$	0.67	0.67	0.33	0.33	0.33	0.67	0.67	0.33	0.67	0.33	0.50
$X_{4}$	1.00	0.67	1.00	0.33	0.67	1.00	0.67	0.67	1.00	0.67	0.77
$X_{5}$	1.00	0.60	0.80	0.60	0.60	0.80	0.60	0.60	0.60	0.80	0.70
$X_{6}$	1.00	1.00	1.00	1.00	1.00	1.00	1.00	1.00	1.00	1.00	1.00
$X_{7}$	1.00	1.00	1.00	1.00	1.00	1.00	1.00	1.00	1.00	1.00	1.00
$X_{8}$	1.00	0.80	0.80	0.80	0.80	1.00	0.80	0.60	1.00	0.80	0.84
$X_{9}$	1.00	0.75	0.75	0.50	1.00	1.00	0.50	0.75	1.00	0.75	0.80
PMC index	8.67	7.49	7.35	6.23	7.07	8.47	6.91	6.62	7.94	7.02	7.38

**Table 7:** Level ranking of 10 representative policies.

Policy	PMC index	Level	Rank
P1	8.67	Reasonable and Complete	1
P2	7.49	Focused	4
P3	7.35	Focused	5
P4	6.23	Weak applicability	10
P5	7.07	Focused	6
P6	8.47	Reasonable and Complete	2
P7	6.91	Focused	8
P8	6.62	Focused	9
P9	7.94	Reasonable and Complete	3
P10	7.02	Focused	7

According to the PMC indices in Table 6, the scores of the 10 smart older adults care system policies are, in descending order, P1 > P6 > P9 > P2 > P3 > P5 > P10 > P7 > P8 > P4. In terms of the rating scale, the average PMC index value of the 10 representative policies is 7.38, which belongs to the level of “Focused,” among which the PMC indexes of 4 policies, namely P1, P2, P6, and P9, are higher than or equal to the average value. Among these 10 policies, the PMC indices of the two selected central policies are higher than the average value, compared with the remaining eight local policies, only two of which are higher than the average value, indicating that the central policies are better than the local policies as a whole, and that the overall level of the smart older adults care system policies is better, with a certain degree of scientific validity and feasibility, although improvement is still needed in some aspects.

Among provincial and municipal policies, the mean value of the four policies at the provincial level is 7.28 and the mean value of the four policies at the municipal level is 7.12, indicating that provincial policies are better than municipal policies overall. Shandong Province and Shanghai Municipality have higher scores for their older adults policies, at 8.47 and 7.94, respectively. Zhejiang Province and Tianjin Municipality have lower scores for their pension policies, 6.23 and 6.62 respectively, and their formulation in regards to smart older adults care system policies needs to be improved. The PMC index scores vary greatly among provinces and cities, and the reason for this difference may be the different degrees of aging and economic development in each province and city.

**Figure 2:**
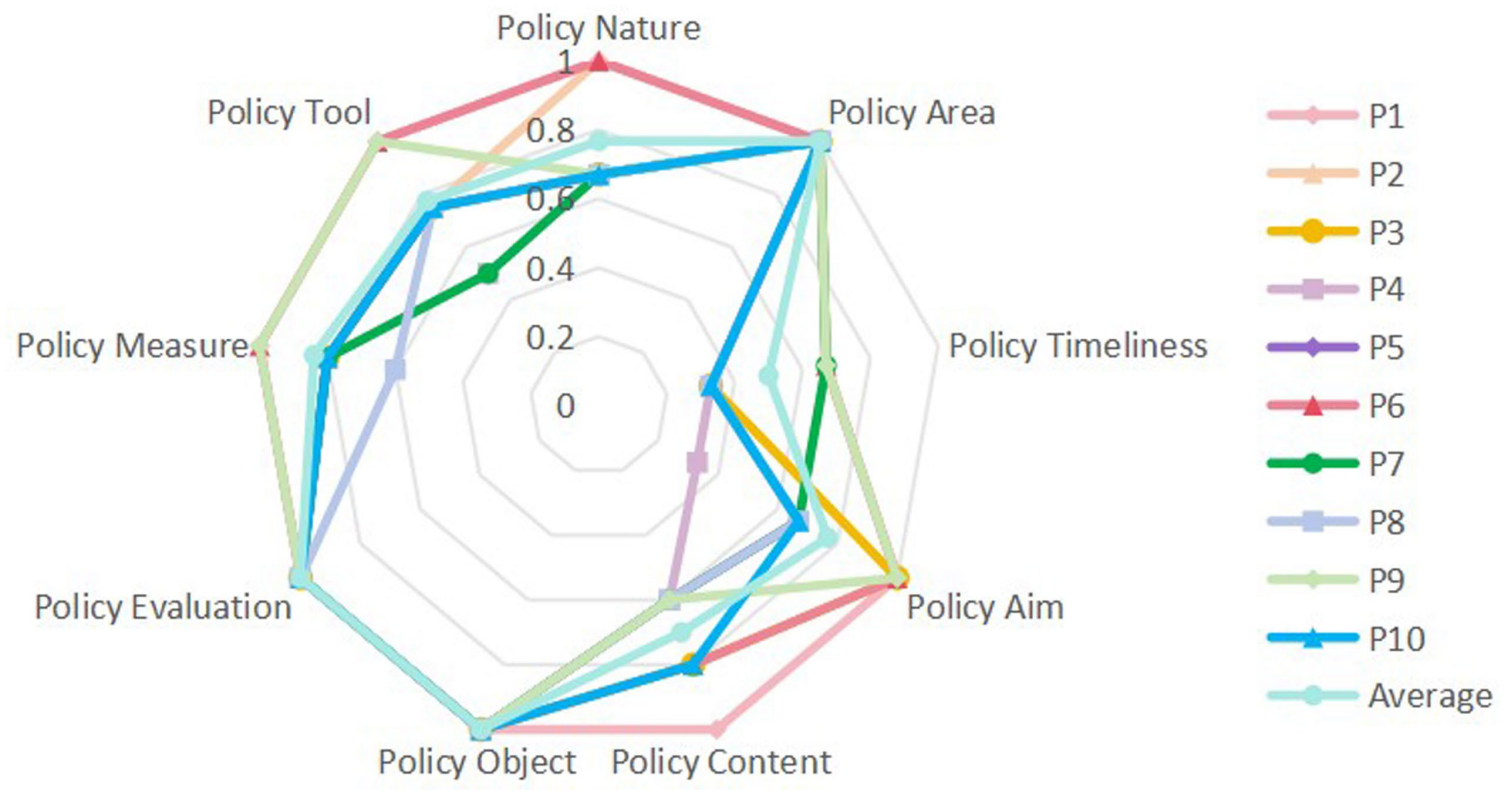
Radar map.

In order to compare the 10 policies horizontally, we make a radar chart based on the data in Table 6, as shown in Figure 2. Its intuitive and clear display of the scores and concavity of the 9 primary variables of the 10 policies helps to identify the strengths and weaknesses of the smart older adults care system policies, and provides direction for the subsequent optimization of the policies.

The provinces and municipalities are different and there are some differences in the development of various aspects of smart older adults care system policy. According to the mean value of the PMC index score for the primary variable $X_{1}$, the mean value of policy nature is 0.77, the selected smart older adults care system policies involve regulatory and guidance elements, and most of the policies lack the prediction of future development outcomes of smart older adults care system. $X_{2}$, $X_{6}\;$ and $\;X_{7}\;$ have the highest mean scores of 1, indicating that all 10 smart older adults care system policies involve economic, medical and scientific and technological areas, taking into account the multiple dimensions of smart older adults care system in social development; the policies involve a wide range of objects, giving full play to the role of each policy subject in smart older adults care system; the policies are based on a sufficient basis, the planning content is rich and the program is scientific, and the overall framework of the policies does not have any major flaws, and it has certain scientific and feasibility, and the layout of the policies is comprehensive. The mean value of $\;X_{3}$ is 0.5, the lowest score, and many policies do not combine long-term, medium-term and short-term goals, indicating that most smart older adults care system policies tend to set short-term goals at the expense of long-term goals. The mean value of $\;X_{4}$ is 0.77 and scored the same as $\;X_{1}$. All 10 policy aims are related to the optimization of service provision, indicating that the comprehensiveness of the objectives is not taken into account in policy formulation, but there are shortcomings in product development and promotion. The mean value of $\;X_{5}$ is 0.7. The policies all involve technical training, government subsidies and smart devices, but lack relevant requirements in application pilots and cybersecurity, especially in cybersecurity, which is only covered by one central-level policy, indicating that the information cybersecurity dimension has not been sufficiently considered in the formulation of smart older adults care system policies and needs to be strengthened. The average value of $\;X_{8}$ is 0.84, ranking second, indicating that the formulation of policy measures is more comprehensive overall. However, in terms of institutional reform, local policymakers still need to formulate a more comprehensive program. The mean value of $\;X_{9}$ is 0.8, with the second highest score after $\;X_{8}$. The selected smart older adults care system policies focus on coercive, innovative and market-based tools, but in terms of cooperative tools, only three policies are involved and lack application.

### 4.3 PMC surface diagram analysis of 10 representative policies

This paper entered the scores for the level 1 variables in a 3 × 3 matrix into the data analysis software and used MATLAB to plot the PMC surfaces of the 10 policies, as shown in Figures 3–12.

**Figure 3:**
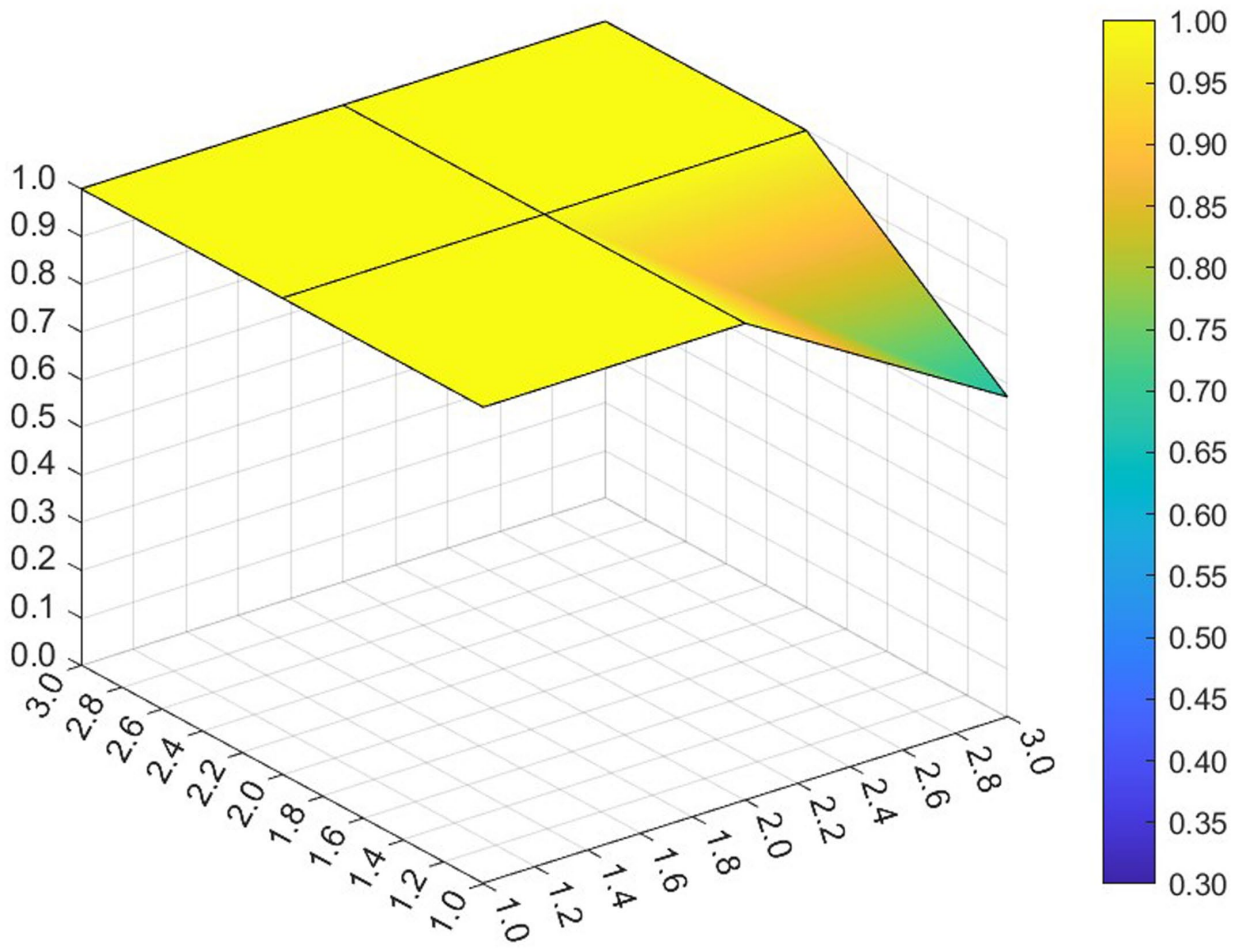
A PMC surface diagram of P1.

**Figure 4:**
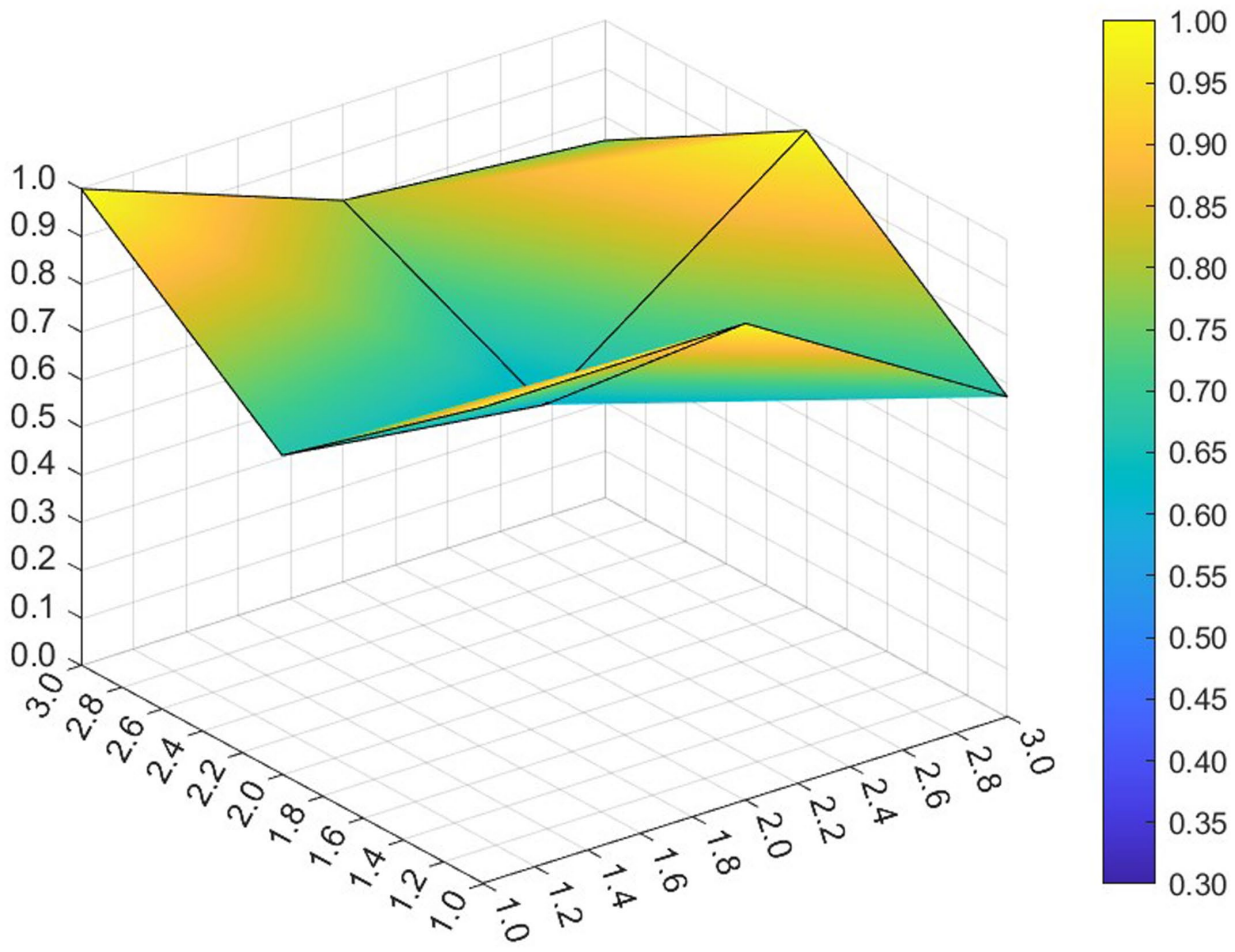
A PMC surface diagram of P2.

**Figure 5:**
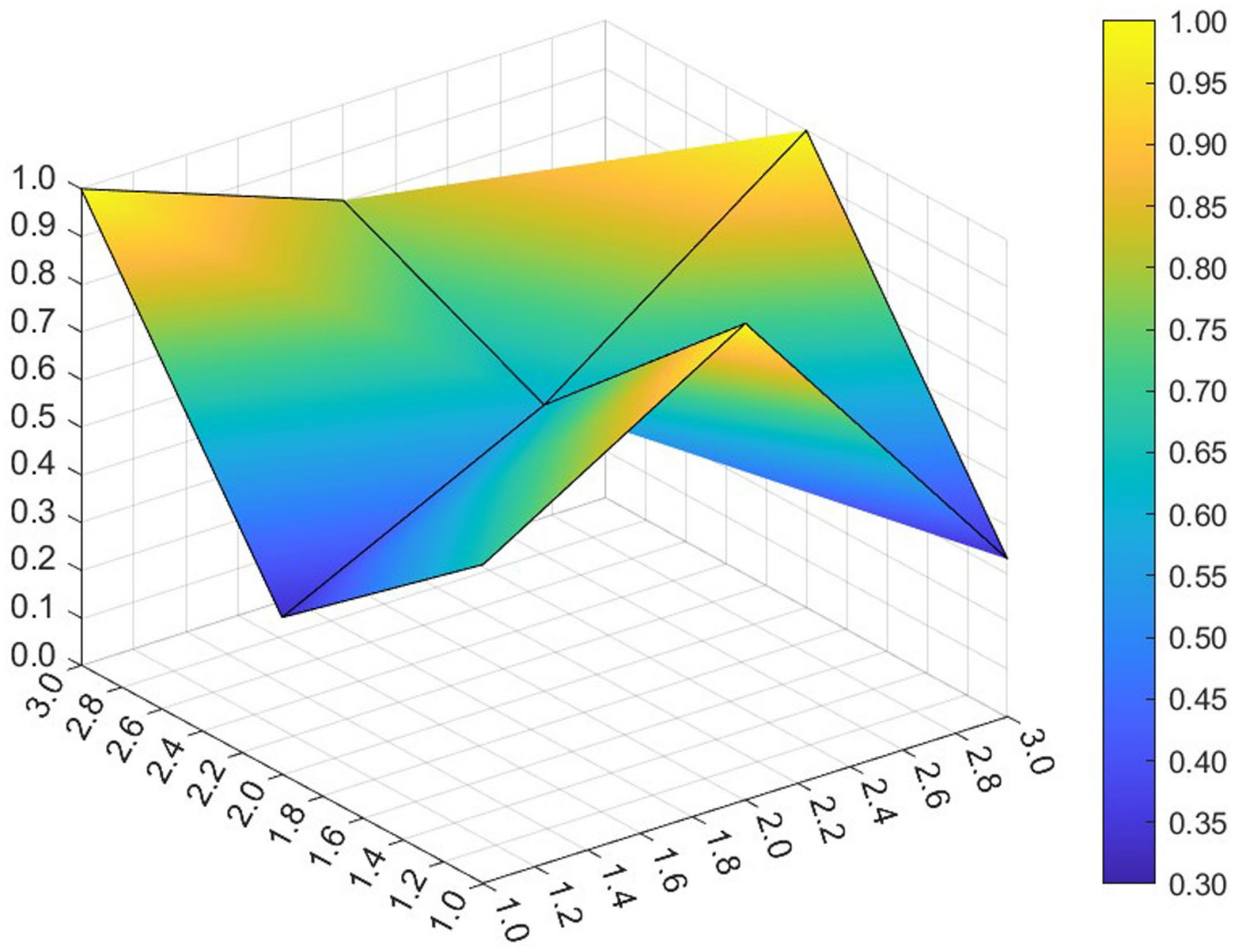
A PMC surface diagram of P3.

**Figure 6:**
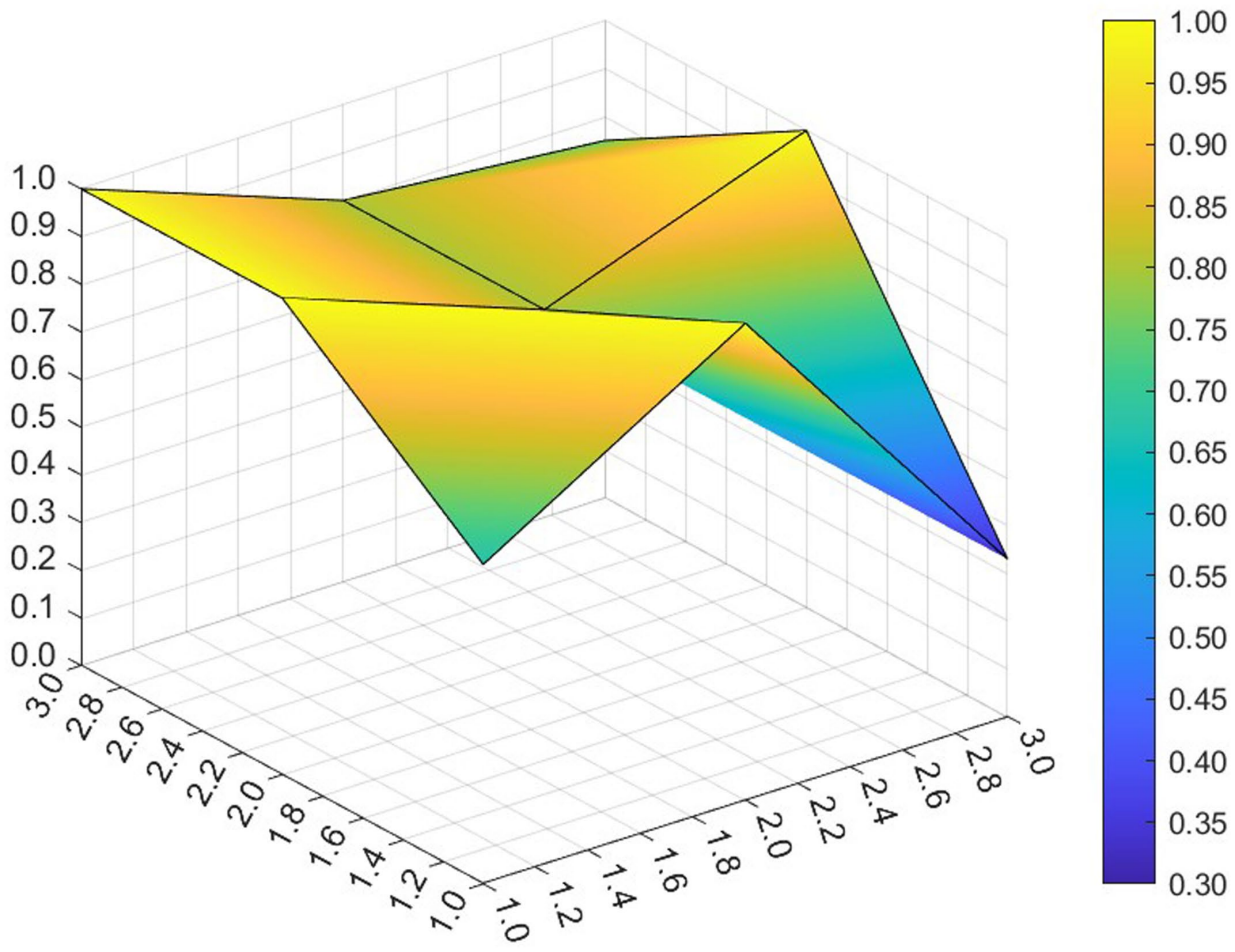
A PMC surface diagram of P4.

**Figure 7:**
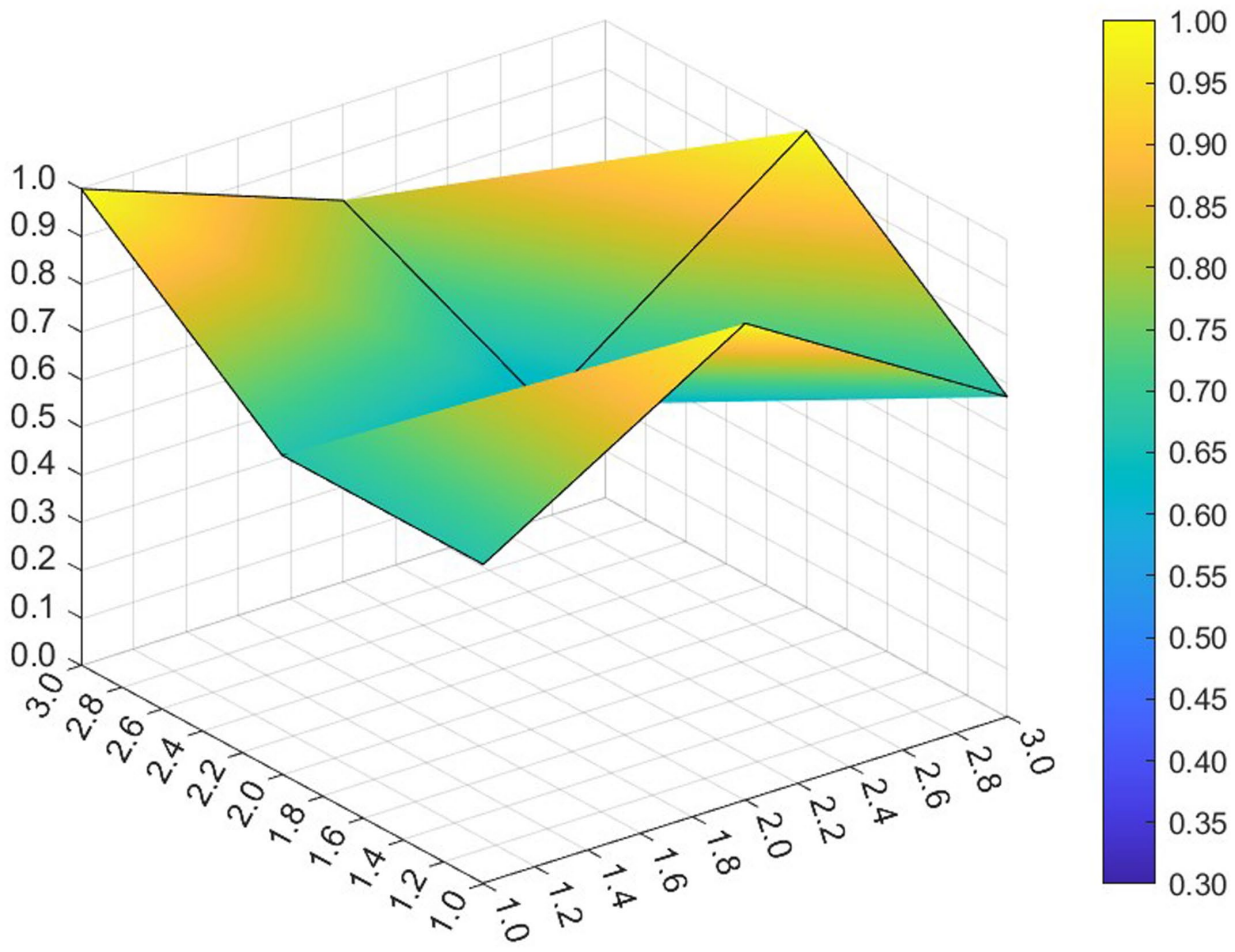
A PMC surface diagram of P5.

**Figure 8:**
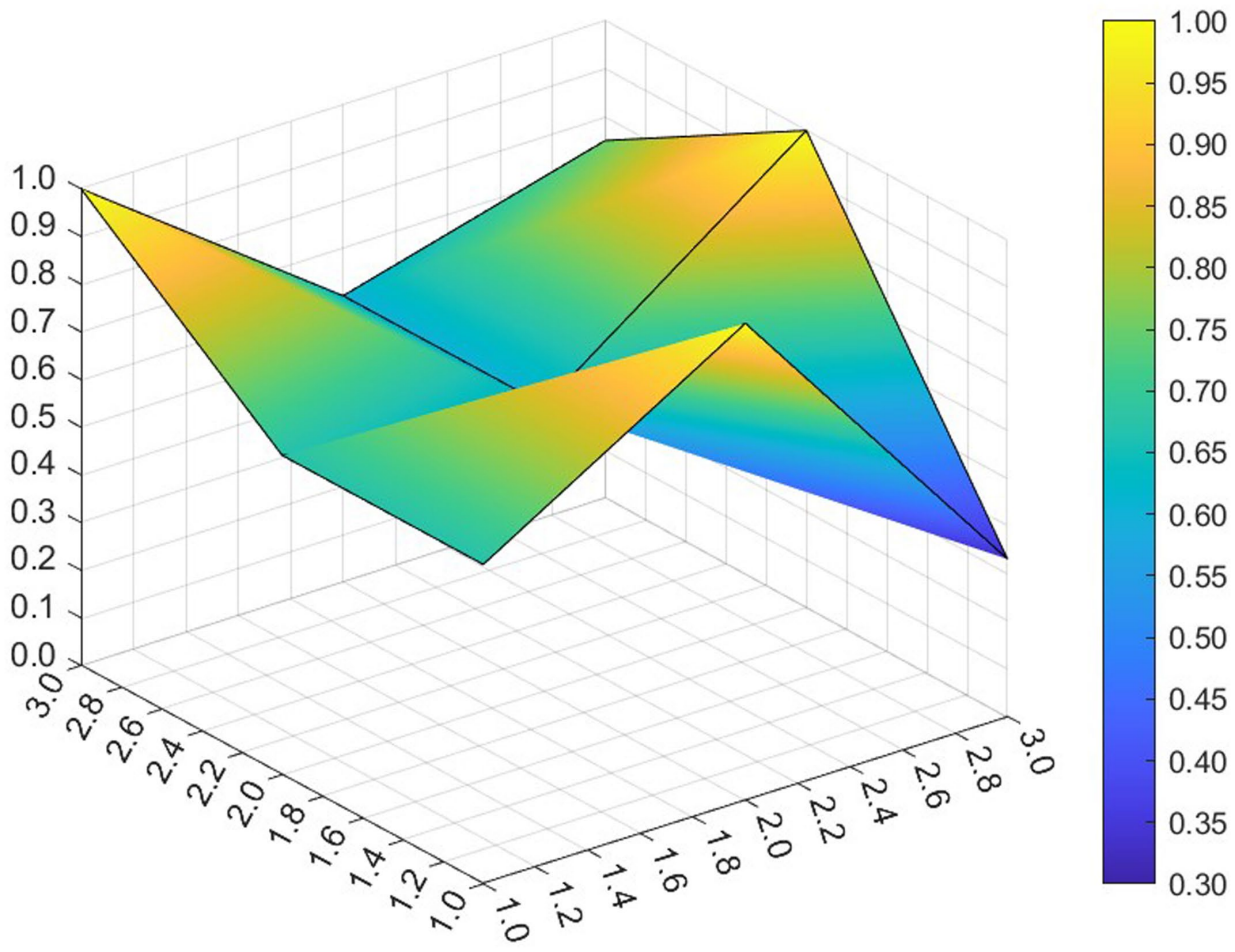
A PMC surface diagram of P6.

**Figure 9:**
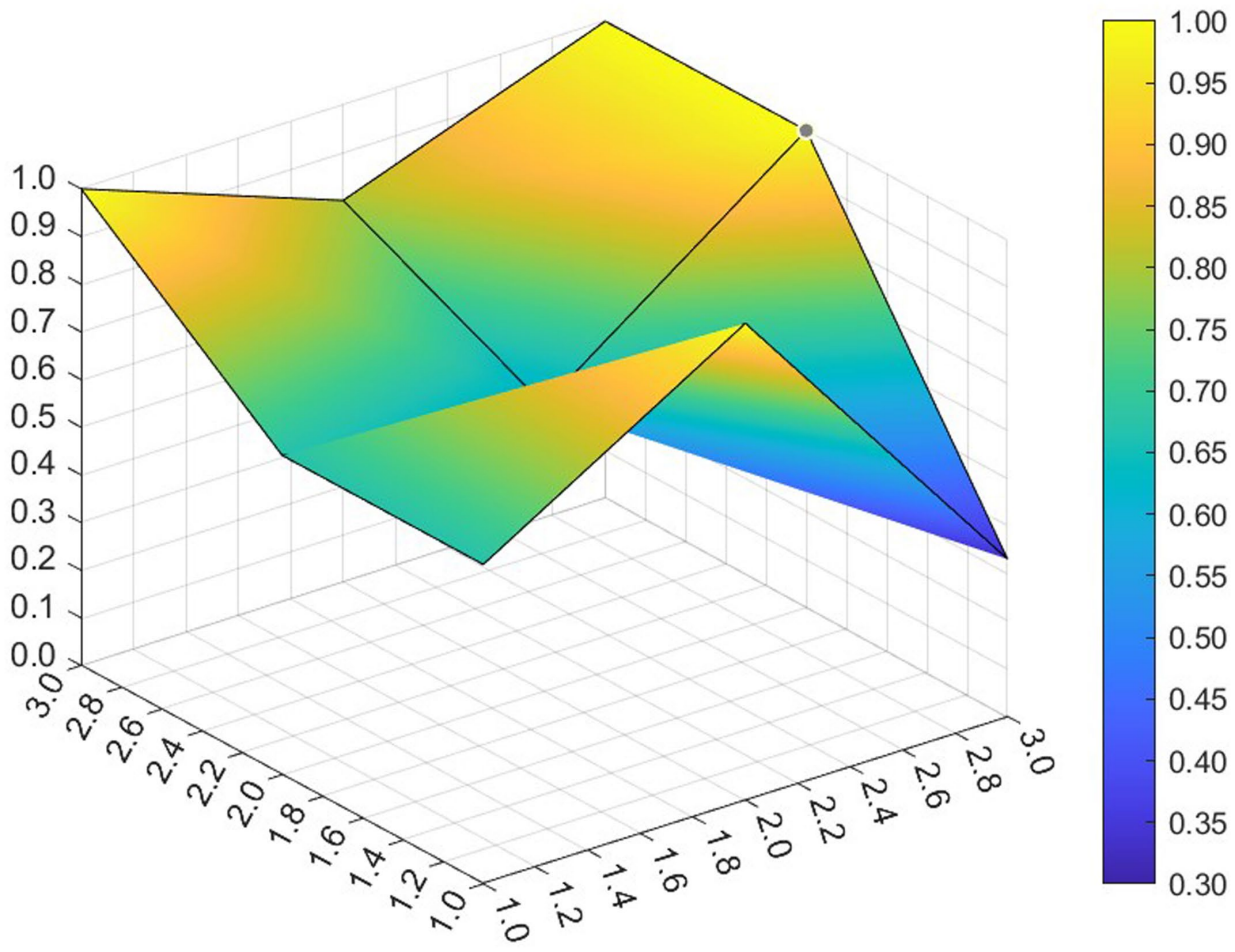
A PMC surface diagram of P7.

**Figure 10:**
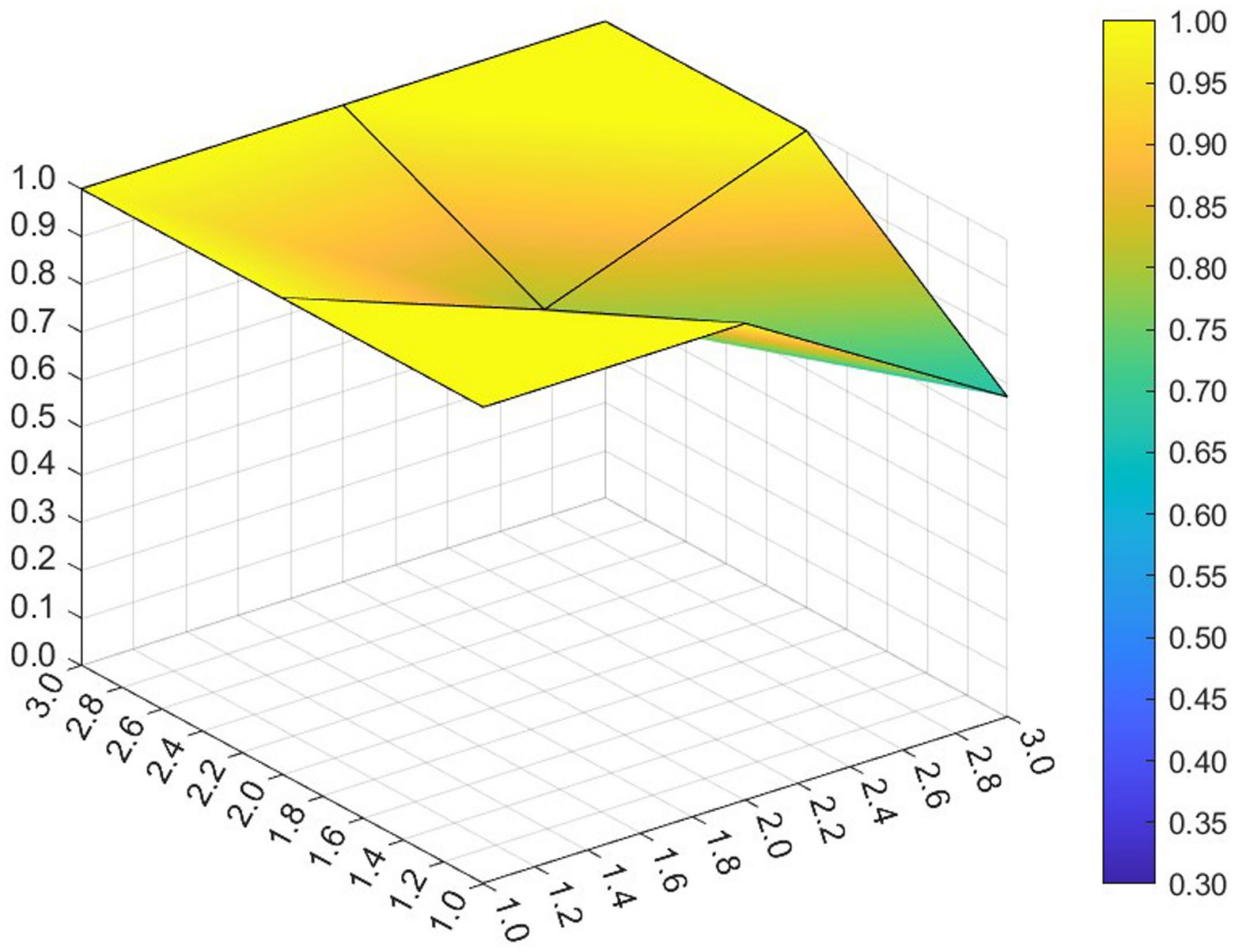
A PMC surface diagram of P8.

**Figure 11:**
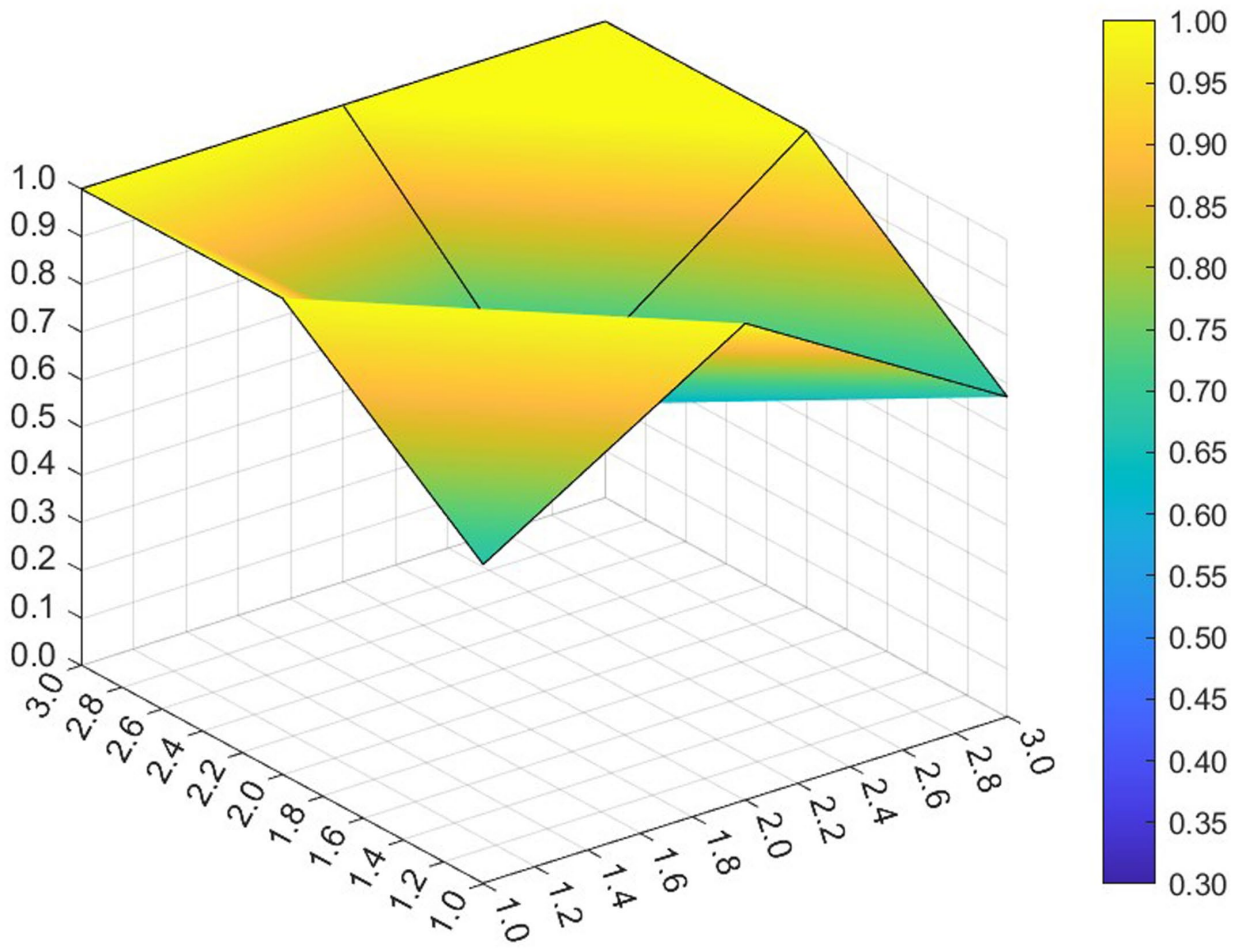
A PMC surface diagram of P9.

**Figure 12:**
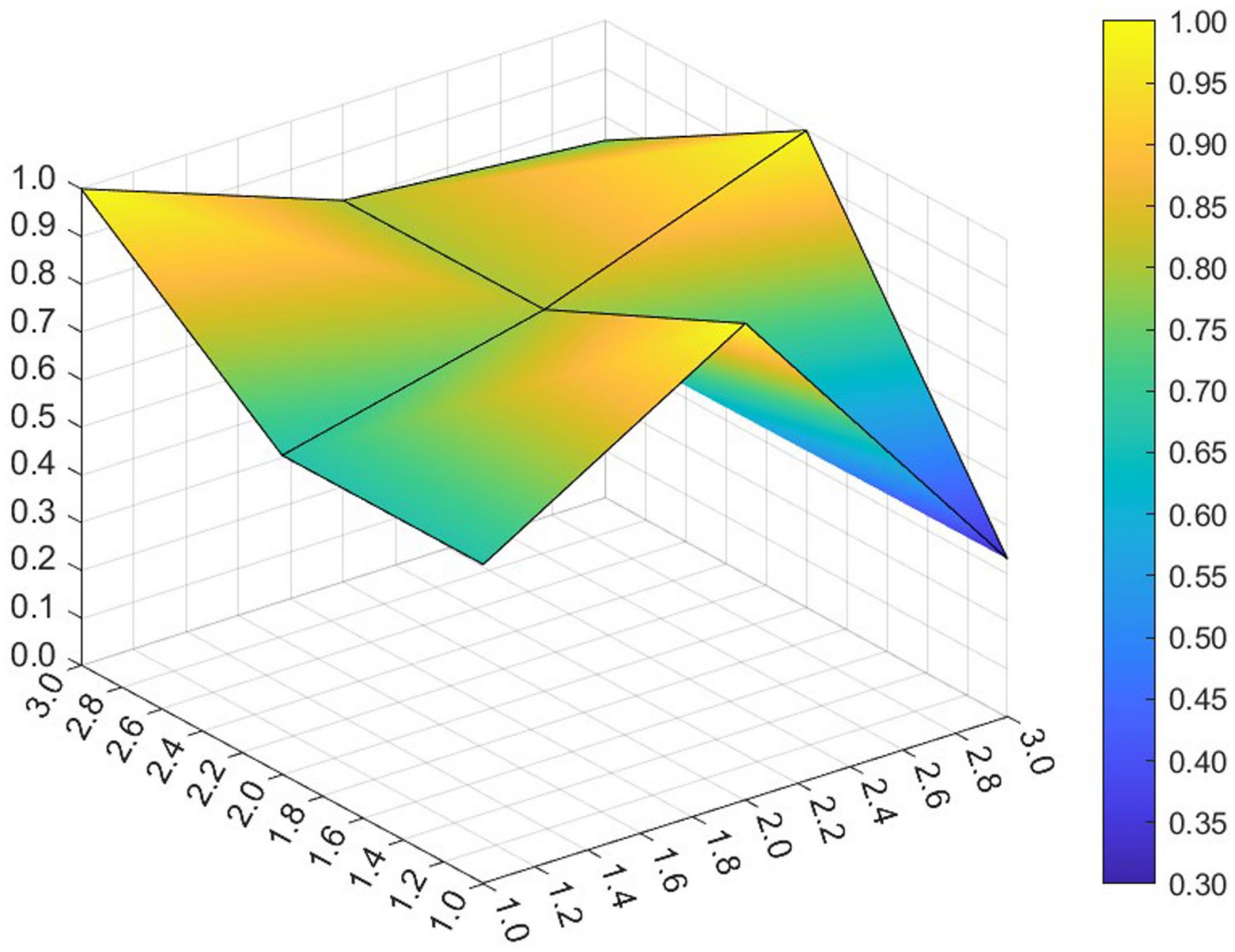
A PMC surface diagram of P10.

The different colors in the graph represent the corresponding scores for each level of the indicator. The lower the score, the darker the color of the indicator on the graph, the greater the degree of depression. Through the trend of the surface’s ups and downs, it can compare the differences and advantages and disadvantages of each policy. In the surface graph, the bottom$\;\;X_{1}$ to $\;X_{9}$ matrix-level variables are mirrored by the bumps and concave points on the surface, with the points closer to the highest point indicating a higher degree of internal consistency within the policy (42).

Policy P1 is a centralized policy issued by the State Council, with a PMC index of 8.67, ranked first, and assessed as “Reasonable and Complete,” with the best overall performance. The policy was planned in the context of the 14th Five-Year Plan period, when China began a new journey to build a modern socialist country in an all-round way, and the CPC Central Committee elevated actively responding to population aging to the level of a national strategy. The scores of the nine primary indicators of this policy are all higher than the average value, and the content coverage is comprehensive, scientific and reasonable. It has certain reference significance. As an overall policy at the national level, it is recommended that long-term, medium-term and short-term goals be combined in terms of the policy’s timeliness, so as to better define the policy’s goals at each stage and improve efficiency. Subsequently, 14 provinces have gradually introduced corresponding local plans and implementing regulations based on the national master plan for the older adults and local needs for the older adults, which is consistent with the results of this study.

Policy P2 has a PMC index of 7.49, ranking 4th, and is assessed as “Focused.” The policy gives full play to the roles of regulation and guidance, combines short-term and medium-term goals, and involves a number of neighborhoods and targets, which is scientific to a certain extent. However, this policy is not perfect in the formulation of $X_{4}\;$ Policy Aim, $X_{5}\;$ Policy Content, $X_{8}\;$ Policy Measure and $X_{9}\;$ Policy Tool, and there is some room for improvement. If there is a need for policy improvement, the order of improvement suggestions can be made with reference to the difference between the variable and the mean value from the largest to the smallest, so the suggested order of improvement is$\;X_{5}$ →$X_{4}$ →$X_{8}$ →$X_{9}$. This ordering is not absolute and should be adjusted and improved in the light of the specific circumstances of the policy.

Policy P3 has a PMC index of 7.35, ranking 5th and assessed as “Focused.” In this policy, the score of 5 primary variables are greater than or equal to the average, but in $X_{1}$ Policy Nature, $X_{3}$ Policy Timeliness and $X_{9}$ Policy Tool, their scores are below average. Specific performance is that the policy timeliness is relatively single, mainly with short-term aims. It needs to strengthen the application of pilots and the use of cooperative policy tools. As a whole, according to the degree that each indicator is less than the mean, the suggested path of improvement is as follows$\;X_{3}$ →$X_{1}$ →$X_{9}$ →$X_{8}$.

Policy P4 has a PMC index of 6.23 and ranks 10th, with an assessment level of “weak applicability,” the lowest ranking and the worst performance among all policies. P4 is more comprehensive in terms of $X_{2}\;$ Policy Area, $X_{6}\;$ Policy Object, and $X_{7}$ Policy Evaluation. However, the score on $X_{1}\;$ Policy Nature, $X_{3}\;$ Policy Timeliness, $X_{4}\;$ Policy Aim, $X_{5}\;$ Policy Content, $X_{8}\;$ Policy Measure, $X_{9}$ Policy Tool are all lower than the mean. Among the policies, the Zhejiang Civil Affairs Department launched the digital civil affairs project and formulated a five-year plan, while the short-term plan lacks a corresponding description. According to the extent that each indicator is less than the mean, the suggested improvement path is $\;X_{4}$ →$X_{9}$ →$X_{3}$ →$X_{1}$ →$X_{5}$ →$X_{8}$.

Policy P5 has a PMC index of 7.07 and is ranked 6th, with an assessment rating of “Focused.” The policy’s $X_{2}\;$ Policy Area, $X_{6}$ Policy Object, $X_{7}$ Policy Evaluation, $X_{9}$ Policy Tool scored full marks. It clarifies the responsibilities of all parties in older adults care services, highlights the government’s position of “covering the bottom line and ensuring the basics,” supports the participation of social forces, emphasizes the obligations of families in older adults care, and gives full play to the roles of all the main parties. However, in $X_{1}\;$ Policy Nature, $X_{3}\;$ Policy Timeliness, $X_{4}$ Policy Aim, $X_{5}$ Policy Content and $X_{8}\;$ Policy Measure need to be optimized. According to the extent to which each indicator is less than the mean, the suggested improvement path for P5 is $\;X_{3}$ →$X_{4}$ →$X_{1}$ →$X_{5}$ →$X_{8}$.

The PMC index of Policy P6 was 8.47, ranking 2nd, with an assessment level of “Reasonable and Complete,” the highest among the selected provincial policies. Eight of the primary variables have scores greater than or equal to the mean value. The policy combines medium-term and short-term goals, is well-founded and reasonably complete, covers application pilots, technical training, government subsidies, smart devices, etc., and involves measures such as institutional reforms, financial support, talent cultivation, legal safeguards, and assessment, etc. It is a comprehensive application of the policy tools and gives full play to its utility in smart older adults care system. However, it lacks application of requirements in cybersecurity-related aspects. It is suggested that the policy should strengthen the personal information and privacy security of the older adults in the process of using digital and informationized networks.

The PMC index of Policy P7 is 6.91, ranking 8th and assessed as “Focused.” In this policy, its $X_{1}\;$ Policy Nature, $X_{4}\;$ Policy Aim, $X_{5}\;$ Policy Content, $X_{8}\;$ Policy Measure and $X_{9}\;$ Policy Tool score below the mean. The Regulations provide a detailed list and categorization of government and departmental responsibilities, service provision, service facilities, etc. It combines long-term and short-term objectives and focuses on policy continuity and stability, but it does not articulate policy expectations or give a description of expected future outcomes. In $X_{8}\;$ Policy Measure, there is still room for improvement. There are fewer requirements for the application of intelligentized products in the process of home-based older adults care services, involving less product research and development, technological innovation or industrial integration innovation, and according to the degree to which each indicator is smaller than the mean value, the suggested improvement paths are $\;X_{9}$ →$X_{5}$ →$X_{4}$ →$X_{1}$ →$X_{8}$.

Policy P8 has a PMC index of 6.62 and ranks 9th, with an assessment rating of “Focused.” The Tianjin Regulations on the Promotion of Older Adults Services is the first local regulation to promote the development of older adults services in China, and the policy was newly revised in 2020 to meet the actual needs of the older adults. The policy adds medical and nursing services, strengthens support for home-based older adults care, covers a wide range of neighborhoods, encourages innovative industrial integration, and covers government subsidies, education for the older adults, and the application of intelligent equipment, but its $X_{8}\;$ Policy Measure differs greatly from the mean value, and have the lowest scores among the selected municipal policies, covering a smaller scope. Most of them aim at short-term programs, provide methods and guidance, and do not reflect the expected final goals and effects, leaving much room for improvement. Enhancing the effectiveness of the policies requires a focus on local objectives, taking into account the overall context. According to the extent to which each indicator is less than the mean, the suggested path for improvement is $\;X_{8}$ →$X_{3}$ →$X_{4}$ →$X_{5}$ →$X_{1}$ →$X_{9}$.

Policy P9 has a PMC index of 7.94, ranking third, with an assessment grade of “Reasonable and Complete,” the highest among municipal policies. The Ordinance covers almost all aspects of older adults care services and is the “basic law” leading the development of older adults care services in the city. The policy covers more than neighborhoods, combines long-term and short-term goals, has clear objectives, covers multiple participants, is well-founded, has feasible policies, and has comprehensive measures, including government subsidies, smart devices, and technical training, to help the older adults cross the digital divide. Geographically, the integrated development strategy for the older adults in the Yangtze River Delta region promotes the development of the older adults industry in Shanghai. In addition, the policy utilizes more comprehensive tools, involves cooperation with international markets, and gives full play to the allocation of market resources. However, the policy has deficiencies in information technology security, and if improvements are needed, it is recommended that the security management of cyberspace be strengthened.

Policy P10 has a PMC index of 7.02, ranking 7th, with an assessment grade of “Focused.” It is the first local regulation in the province of Guangdong that focuses on regulating home-based older adults care services, and it is also the first time that Zhuhai has carried out legislation in the field of older adults care. The policy in $X_{2}\;$ Policy Area, $X_{5}$ Policy Content, $X_{6}$ Policy Object and $X_{7}$ Policy Evaluation score high. However, $X_{1}$ Policy Nature, $X_{3}$ Policy Timeliness, $X_{4}$ Policy Aim, $X_{8}$ Policy Measure and $X_{9}$ Policy Tool could be improved. The policy prescription is single, and it is suggested to add more specific phased goals. As a special economic zone, international cooperation should be considered in the process of policy formulation to promote economic development. According to the degree that each indicator is less than the mean, the suggested improvement path is $\;X_{3}$ →$X_{4}$ →$X_{1}$ →$X_{9}$ →$X_{8}$.

## 5 Discussion

Through the analysis of PMC index of 10 representative smart older adults care system policies, this study reveals the key characteristics and deficiencies of the current policy system, which are discussed as follows:

1. Structural imbalance in Policy Design.

The PMC index analysis reveals significant structural imbalances at the macro level of China’s policy framework. Specifically, five, six, and seven policies, respectively, scored below average in policy effectiveness, objectives and content, as well as policy instruments and tools. This clearly indicates that the smart older adults care system policy, a core initiative addressing population aging, still requires improvements in top-level design regarding systematicness, foresight, and operational feasibility. Urgent enhancements are needed in temporal coordination, objective setting, content refinement, innovative measures, and tool adaptability.

2. Insufficient coherence and long-term orientation in target planning.

Most policies analyzed lean toward short-term goals, showing a marked lack of medium-to-long-term strategic planning. This approach often sacrifices long-term welfare for immediate results, making it difficult to establish sustainable policy chains. Given that the smart older adults care system policy is a crucial component of China’s national aging-related development plans during the 14th Five-Year Plan period and beyond, its implementation must align closely with national planning cycles. Furthermore, adjustments and optimizations should be made dynamically based on regional development realities.

3. Disconnection between product development and demand satisfaction.

Analysis reveals a significant gap between product development efforts and the diverse, differentiated, and personalized needs of older adults users. Many current smart older adults care products fail to deeply align with actual requirements, resulting in a “functional-demand mismatch” (43). The core challenges lie in two aspects: First, while seniors generally prioritize ease of use, most smart devices feature complex operations that create a severe “digital divide.” The lack of unified standards and widespread adoption further exacerbates cognitive barriers and user resistance (43). Second, constrained by market maturity, fragmented data, user acceptance, and resource allocation, products often prioritize visual appeal over practical functionality. This disconnect stems from insufficient understanding of older adults users’ needs and misguided R&D priorities.

4. Security risks and governance gaps in the digital empowerment era.

Amid the accelerated integration of “digital intelligence empowerment,” cybersecurity risks have emerged as a critical vulnerability for the development of the smart older adults care system. The evaluation metrics reveal that smart older adults care system policies demonstrate the weakest implementation in the “cybersecurity” dimension, with only the P1 policy explicitly addressing older adults privacy protection. Key vulnerabilities include: First, smart device systems extensively collect sensitive data from seniors. When publicly available older adults care service data is subjected to algorithmic discrimination or “black box operations” driven by capital interests, it becomes highly vulnerable to privacy breaches and fraud (44). Second, the internet’s open architecture makes it susceptible to hacker attacks and malware threats, posing risks of technology abuse (44). These challenges necessitate policymakers to embrace technological dividends while maintaining heightened vigilance and implementing systematic safeguards against accompanying security and ethical dilemmas.

5. Implementation mechanism deviation: overreliance on top-down models.

The core safeguards of the current smart older adults care system policy primarily focus on financial support, talent development, legal protection, and performance evaluation. However, the existing performance assessment mechanism predominantly exhibits a “top-down” characteristic, which contradicts the essential nature of older adults care supply–demand perception that should follow a “bottom-up” approach (45). While the “government-led, centrally coordinated” model strengthens planning consistency, it may lead to excessive reliance on government intervention, suppress market vitality and social participation, weaken grassroots enthusiasm, and create information barriers and decision-making power imbalances among stakeholders. In contrast, the “village” volunteer model in rural communities of the United States—characterized by bottom-up participation and multi-stakeholder engagement, provides valuable insights for addressing diverse needs and stimulating grassroots dynamism (46). China should explore a collaborative governance structure combining “top-down guidance” with “bottom-up responsiveness, “shifting roles from “government-led” to “government-guided.” This requires incorporating public participation into performance evaluation systems, establishing engagement platforms, and strengthening assessment mechanisms to ensure demand fulfillment.

## 6 Conclusion and optimization path

### 6.1 Conclusion

This study focuses on smart older adults care system and selects 10 representative central and local policies from 2019 to 2024 as the basis for constructing the policy evaluation index system of smart older adults care system. Through text mining and PMC index, this study conducts quantitative evaluation on the 10 policies. The research conclusions are as follows:

China’s smart older adults care system policy has established a systematic framework at the macro-level, demonstrating notable achievements in broad domain coverage and multi-stakeholder collaboration mechanisms. However, the policy system still faces three structural shortcomings: (1) Overemphasis on short-term goals without aligning with medium-to-long-term roadmaps like the 14th Five-Year Plan. (2) Disconnection between smart product development and older adults care needs, coupled with near-absence of cybersecurity protection mechanisms. (3) Overreliance on top-down mandatory tools while neglecting bottom-up feedback mechanisms and international cooperation potential. Future policy optimization should focus on layered goal alignment, demand-driven innovation, integrated security governance, and multi-stakeholder collaboration to transition smart older adults care system policies from “government-led” to “precision-responsive.” Based on this, this paper proposes the following optimization pathways for further improving the smart older adults care policy system.

### 6.2 Optimization path

#### 6.2.1 Establish a hierarchical goal planning system, optimize the policy structure, and clarify the implementation standards

As smart older adults care practices deepen, governments should strengthen systematic planning of policy frameworks, clarify long-term objectives across sectors, and coordinate short-to-long-term development rhythms to drive high-quality growth. It is recommended that all national-level smart older adults care system policy documents explicitly include 5-year mid-term goals and 15-20-year vision blueprints, while requiring provincial and local governments to formulate 3-year rolling implementation plans to ensure seamless alignment with national planning cycles. Provincial authorities should break down national targets into regional-specific components when formulating policies, regularly assess the compatibility between local policies and higher-level plans as well as regional realities, and issue adjustment guidelines. When developing smart older adults care system policies, both central and local governments should refine objectives into actionable sub-goals. For instance, regarding the “enhancing technological and digitalization of older adults services” mentioned in the National Medium-and Long-Term Plan for Actively Addressing Population Aging, specific metrics like smart device adoption rates and improvements in seniors’ information literacy should be clearly defined. Additionally, establishing a unified smart older adults care system service standard system with detailed implementation rules and quantifiable indicators is urgently needed. This will provide clear operational guidance and regulatory benchmarks for policy execution, ensuring standardized and high-quality service delivery.

#### 6.2.2 Enrich policy content, optimize resource allocation, and promote coordinated development

With the advancement of socio-economic and technological development, the needs of the older adults are becoming increasingly diversified. The government’s smart older adults care system policy must not only address these varied demands but also account for regional disparities to avoid a one-size-fits-all approach. Authorities should prioritize establishing a technology-driven, demand-oriented, and equitable policy framework. By deeply integrating next-generation technologies like IoT, AI, and big data, clear application scenarios and implementation pathways should be defined for home-based older adults care monitoring, targeted community service delivery, institutional operational efficiency enhancement, and intelligent health management. Detailed technical standards must be established to ensure age-friendly device designs, secure data exchange, and user-friendly operations, thereby enabling smart older adults care system services to truly meet seniors’ multidimensional needs across daily life domains. Additionally, breaking down “information silos” through cross-departmental and cross-level data interoperability will provide precise resource allocation benchmarks. By adopting advanced international smart older adults care system concepts and technologies, China can drive continuous innovation in both content and form of its smart older adults care system policies. Specialized subsidies and social safety nets should be refined for vulnerable groups including the older adults with advanced age, those living alone, individuals with disabilities or cognitive impairments, and low-income seniors. This ensures that technological benefits are “accessible, affordable, and effective,” while fostering collaborative mechanisms between developed and underdeveloped regions to share management expertise and premium digital resources. Ultimately, this will achieve coordinated regional development and enable all seniors to enjoy the convenience, security, and dignity of a smart society.

#### 6.2.3 Strengthen policy supervision to ensure the safety and standardization of smart older adults care system

To ensure the protection of personal information and privacy for seniors using smart older adults care system services, national authorities should expedite the establishment of security regulations. These regulations must specify mandatory technical standards for service providers ‘qualifications, facility configurations, and operational procedures. Strengthening policy oversight requires creating a multi-agency collaborative platform that implements “double random checks with public disclosure” and targeted risk-based supervision through big data analytics. Enterprises violating data collection protocols, information leaks, or service quality standards will face tiered penalties including blacklisting, hefty fines, or license revocation. A national smart older adults care security monitoring system will enable real-time detection of abnormal access attempts and cyberattacks in critical systems, automatically triggering alerts and emergency responses. Service providers must adopt “privacy-friendly” age-appropriate designs, embedding features like “one-click privacy protection” into devices and apps. Older Adults care institutions should conduct cybersecurity training programs to enhance staff and seniors’ awareness of data security responsibilities. This ensures technological benefits are safely delivered within trustworthy frameworks, building a solid foundation for secure smart older adults care development.

#### 6.2.4 Strengthen collaboration among various stakeholders and improve the efficiency of policy implementation

The effective implementation of the smart older adults care system policy requires multi-stakeholder collaboration. To enhance execution efficiency, establishing a collaborative symbiotic mechanism is crucial and this involves clarifying responsibilities, rights, and interests across all parties while building an efficient joint action network. First, the government should take the lead. On one hand, it should establish cross-departmental coordination mechanisms through a unified smart older adults care information platform to achieve dynamic sharing and precise matching of policy information, service resources, fund allocation, and senior citizens’ needs. This ensures seamless collaboration among departments, institutions, and personnel on a single platform, breaking down bureaucratic barriers. On the other hand, the government should lower entry thresholds for tech enterprises and social capital through measures like service procurement, tax incentives, and special subsidies, guiding them to focus on technological innovation, service innovation, and sustainable operational model exploration. Simultaneously, the government can set service standards and performance metrics, introduce independent third-party evaluations, and use assessment results as key references for policy adjustments, fiscal rewards, and entity access. Second, community organizations, social work agencies, volunteer groups, and other social forces should leverage their localized advantages to serve as “last-mile” implementers and coordinators for policy implementation. They are responsible for promoting technology application training, collecting and providing feedback on precise needs, offering personalized emotional support, and fostering grassroots service teams. Families, as key participants, can conveniently access professional support through smart terminals and online platforms to participate in care plan formulation, creating a synergy of government empowerment, social services, and family responsibility fulfillment.
